# Study on the remodeling of distal residual dissection after surgery in patients with type A aortic dissection and Marfan syndrome

**DOI:** 10.1016/j.xjon.2025.06.011

**Published:** 2025-06-26

**Authors:** Junyu Wang, Huiwen Gao, Xuelan Zhang, Kai Tang, Hui Han, Chang Shu, Xiangyang Qian, MingYao Luo

**Affiliations:** a State Key Laboratory of Cardiovascular Disease, Center of Vascular Surgery, Fuwai Hospital, National Center for Cardiovascular Diseases, Chinese Academy of Medical Sciences and Peking Union Medical College, Beijing, China; b School of Mathematics and Physics, University of Science and Technology Beijing, Beijing, China; c Department of Vascular Surgery, Fuwai Yunnan Cardiovascular Hospital, Affiliated Cardiovascular Hospital of Kunming Medical University, Kunming, China; d Department of Vascular Surgery, Central-China Branch of National Center for Cardiovascular Diseases, Henan Cardiovascular Disease Center, Fuwai Central-China Hospital, Central China Fuwai Hospital of Zhengzhou University, Zhengzhou, China

**Keywords:** Marfan syndrome, type A aortic dissection, negative aortic remodeling

## Abstract

**Objective:**

To evaluate the remodeling of the distal aorta and outcomes after aortic surgery for type A aortic dissection (TAAD) in patients with Marfan syndrome and investigate whether morphologic characteristics of the dissection can predict negative remodeling.

**Methods:**

Between 2013 and 2021, we performed total arch with a frozen elephant trunk for 325 patients with Marfan syndrome with DeBakey type I aortic dissection. Mean age was 47.13 ± 7.33 years, and 204 were men (63%). Follow-up was complete in 91.1% (296 out of 325) at a mean of 48.3 ± 13.1 months. Four-year incidence of death was 8.6% and reoperation rate was 10.4%. Negative remodeling was defined as an average growth rate >5 mm/year or >10% at any segment detected by computed tomography angiography.

**Results:**

After surgery, negative remodeling occurred in 19.3% and 26.7% at TAAD follow-up at a mean of 13.6 and 38.3 months, respectively. There were 15.2% (12 out of 79) late deaths and 26.6% (21 out of 79) distal reoperations for those patients. The positive remodeling patients share a low rate of late death and distal reoperations of 6.5% (14 out of 217) and 7.8% (17 out of 217) (*P* < .01). Maximal aortic sizes before discharge for negative remodeling patients were 43.2, 35.1, and 32.5 mm, and growth rates were 4.5 ± 1.52, 3.1 ± 1.14, and 3.5 ± 1.33 mm/year at the level of diaphragm, celiac trunk, and renal artery respectively, which is larger and expands more quickly than the patients with positive remodeling (*P* < .01). Distal maximal aortic size (*P* < .01), number of entry tears (*P* = .03), and average entry tears size (*P* = .02) predicted rate of negative remodeling.

**Conclusions:**

Our results suggest that TAAD has a high rate of negative aortic remodeling in patients with Marfan syndrome. Distal maximal aortic size, number of entry tears, and average entry tears size were associated with the rate of negative aortic remodeling in patients with TAAD and Marfan syndrome.

**Figure:**
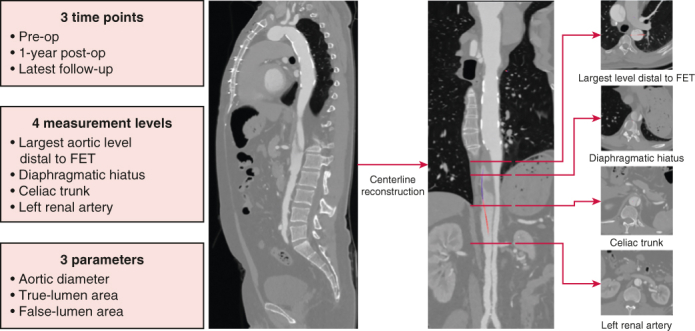
Collecting the morphological and hemodynamic characteristics from CT images.

**Central Message**

Remodeling of residual dissection after surgery was related to the distal maximal aortic size, number of entry tears, and average entry tears size.

**Perspective**

The study showed that maximum diameter of residual dissection and the mean FL area distal to the stent before the surgery were associated with a significantly increased the rate of negative remodeling. Increasing the mean TL area distal to the stent, increased number of intimal tears, and increasing the entry tears size were all associated with positive remodeling.

Marfan syndrome (MFS) is a genetic connective tissue disorder inherited in an autosomal dominant manner, primarily caused by mutations in the fibrillin-1 (*FBN1*) gene. It can affect various organ systems, but the most life-threatening complications are associated with the cardiovascular system, particularly aortic dissection and rupture.^1^ Aortic dissection and rupture are the leading causes of death in patients with MFS, with type A aortic dissection (TAAD) occurring in about 20% to 30% of individuals during their lifetime.^2^

Currently, managing TAAD in patients with MFS remains challenging. Although traditional proximal aortic repair has yielded some positive outcomes, the long-term results are often unsatisfactory. This is due to issues such as residual dissection, patent false lumen (FL), and distal aortic dilation, which increase the risk of distal aortic rupture and the need for further intervention by up to 12 times.^3^, ^4^, ^5^ To address these concerns, total arch replacement with a frozen elephant trunk (FET) has been proposed as a more aggressive repair strategy.^2^^,^^6^, ^7^, ^8^ This approach helps to close off the FL and reduce complications in the distal aorta. The FET technique can expand the true lumen (TL) across the aorta, decrease or stabilize the FL, and stabilize the distal aorta in patients with MFS with TAAD, thereby inducing favorable remodeling in the distal aorta.

At present, surgical intervention is the only effective treatment for TAAD. However, complications often arise postoperatively, including expansion of the distal FL, compression of the TL, critical limb ischemia, and even aortic rupture.^9^ These adverse events are typically linked to negative remodeling, which can lead to a poor prognosis and often necessitate additional treatments, such as further surgery or interventional procedures.^10^^,^^11^ The preoperative diameter of the aorta plays a significant role in aortic remodeling, but prognosis should also consider the condition of the TL and FL.

Previous clinical and bioengineering research has shown that increased inflow and impaired outflow in the FL can significantly raise both mean and diastolic pressures within it. In acute cases, this can lead to collapse of the TL.^12^^,^^13^ Elevated FL pressure may also accelerate aortic growth and increase the risk of aneurysm and rupture. Therefore, the number of entry tears between the TL and FL may be an important predictor of aortic dilation in patients with acute type B aortic dissection. Accordingly, the aim of this study was to identify the characteristics of aortic remodeling after surgery in patients with MFS and to predict the remodeling of distal aortic dissection after surgery through its morphological and hemodynamic characteristics.

## Methods

The Ethics Committees of Fu Wai Hospital of Chinese Academy of Medical Sciences approved this retrospective study (approval No.: 2024-2530; approval date: December 12, 2024). The patient(s) provided informed written consent for the publication of the study data. The patients provided informed written consent for the publication of the study data.

### Patients

Between September 2013 and September 2021, 325 patients with MFS (male predominance: 204 cases [62.8%]) presenting with DeBakey type I aortic dissection involving the aortic arch and extensive descending aortic dissection extending beyond the diaphragm underwent standardized total arch replacement with FET implantation at our center. Mean age was 40.13 ± 13.1 years (range, 24-61 years). The diagnosis of MFS was based on the revised Ghent criteria^14^ or genetic test results are confirmed as MFS. In 2016 Our team performed genetic testing of the FBN1 gene in 39 Chinese probands with Marfan/Marfan-like syndrome and their related family members by Sanger sequencing. In total, 29 pathogenic/likely pathogenic FBN1 mutations, including 17 novel ones, were identified. Since then, we have been conducting genetic testing on patients with MFS for early intervention and treatment.^15^ Patients with a history of open or endovascular repair of the descending aorta (DA) were excluded from this study.

The extent of aortic dissection was to the iliac artery in 185 patients (56.9%), the abdominal aorta in 43 patients (13.2%), and the distal thoracic aorta in 97 patients (29.9%). Before surgery, the presurgical diameters of the aorta at its maximum, at the diaphragm, the celiac trunk, and the left renal artery were 65.4 ± 15.2, 32.6 ± 8.7, 29.3 ± 8.2, and 26.8 ± 7.9 mm, respectively. Among the patients, there were 36 cases of hypertension, 13 cases of diabetes, and 27 cases of coronary heart disease. As baseline data, we selected the following 5 distinct anatomical planes in preoperative imaging studies: aortic sinus plane, maximum diameter plane, diaphragmatic plane, celiac trunk plane, and left renal artery origin plane. The preoperative clinical profiles are listed in Table 1.

**Table 1:** Baseline characteristics in 325 patients with type A aortic dissection and Marfan syundrome

Variable	Result
Age (y)	48.13 ± 7.33
Sex (male/female)	204/121
Follow-up time (mo)	49.3 ± 13.1
Death rate	8.6 (28)
Reintervention rate	10.4 (34)
Hypertension	11.1 (36)
Diabetes	4.0 (13)
Coronary heart disease	8.3 (27)
Family history	3.1 (10)
Preoperative distal maximal aortic size (mm)	65.4 ± 15.2
Preoperative aortic size at different levels	
Aortic sinus	49.8 ± 9.3
Diaphragm	32.6 ± 8.7
Celiac trunk	29.3 ± 8.2
Renal artery	26.8 ± 7.9

Values are presented as mean ± standard deviation or % (n).

### Surgical Technique

Standardized total arch replacement with FET implantation was systematically performed in all patients with MFS presenting with TAAD. The surgical protocol involved median sternotomy with establishment of cardiopulmonary bypass and unilateral antegrade cerebral perfusion via right axillary artery cannulation. Aortic exposure was achieved through longitudinal incision of the ascending aorta extending to the transverse arch, terminating at the origin of the left subclavian artery where circumferential transection of the descending aorta was performed. A stented graft is inserted into the TL of the descending aorta and deployed to compress the FL and enlarge the TL at the same time. The stented graft implanted in the descending aorta is 12 cm in length. The diameter of the stented graft should also be tailored to different aortic morphology. In all dissection patients, we use a 24- to 28-mm stented graft, which is within the normal anatomic ranges of the Chinese population and can often sufficiently enlarge the TL and fit the 4-branched graft. So the stented graft can be anastomosed to the graft in an end-to-end fashion.

Arch vessel reconstruction was performed after distal anastomosis. To optimize cerebral protection, sequential reconstruction commenced with the left common carotid artery anastomosis. Proximal graft-to-aortic root anastomosis was then completed using continuous polypropylene suturing technique. After the ascending aorta was thoroughly de-aired, we restored coronary perfusion and cardiac activity. Finally, the left subclavian and innominate arteries were sutured in turn to the branches of the 4-branched graft in an end-to-end fashion.

The descending aortic rupture of the patients we enrolled was stable before surgery and no treatment was required during the intraoperative evaluation. No early thoracic endovascular aortic repair was performed for patients with larger tears in descending thoracic aortic at the same time.

### Clinical and Imaging Follow-up

Computed tomographic angiography (CTA) of the entire aorta was performed at 3 months, 1 year, and annually henceforth to assess the FL, TL, maximal aortic size and growth rate, and complications (eg, distal new entry, endoleak, and migration). The CTA images at 1-year follow-up were selected as the first follow-up, and the most recent image review results were selected as the second follow-up. CTA measurements and analyses were performed at 4 aortic levels: maximum aortic diameter level, diaphragm level, celiac trunk level, and left renal artery level. The statuses of the FL were classified as completely thrombosed (obliterated) if no flow was present, partially thrombosed if both flow and thrombus were present, and patent if flow was present in the absence of thrombus.

The maximum aortic diameter was measured from the outer contours of the aortic wall in the axial plane. TL and FL area were measured though the contour of the intimal flap. The segmental aortic growth rate (in millimeters per year) was assessed in patients who had undergone 2 or more CT scans postoperatively with an interval of at least 3 months apart and calculated by dividing the diameter differences between the first and last CTAs by the interval in-between. Clinical and radiologic follow-up was complete in 91.1% (296 out of 325) for a mean duration of 49.3 ± 13.1 months.

### Study End Points

The primary clinical end points included late death and distal aortic reoperations. Late death referred to all-cause mortality occurring during follow-up, excluding operative mortality (which was defined as death within 30 days of surgery or before final hospital discharge, including transfers). Distal aortic reoperation included any open or endovascular reintervention on the distal aorta. Open or endovascular reintervention was indicated for patients with a distal maximal aortic size >50 mm. Earlier reintervention would be recommended for patients with a positive family history, distal new entry, or new onset of refractory chest pain. Negative remodeling was defined as a maximal distal aortic diameter of an average growth rate >10% or 5 mm/year at any aortic segment as detected by CTA during follow-up.

### Statistical Analysis

Statistical analysis was performed using SPSS for Windows 22.0. Data are expressed as mean ± standard deviation or number (percentage) and were compared using the Student *t* test or Pearson χ^2^ test for normal distributions, and the Mann-Whitney *U* test for abnormal distributions, as appropriate.

Risk factors for distal aortic dilatation (binary), reoperation, and late death were identified with the Cox regression model. Variables considered in multivariate analyses included acuity, age, gender, hypertension, preoperative maximal diameter of the distal aorta, patent FL in the DA, growth rate, and FET diameter.

## Results

### Operative Mortality and Reoperation

For the whole cohort, survival was 91.4% at 3 years (Table 1). The cause of death was multiorgan failure in 11 patients (3.4%), heart failure in 8 patients (2.5%), stroke in 6 patients (1.8%), and distal aortic rupture in 3 patients (0.9%).

Complications included stroke in 8 patients (2.5%), visceral and lower limb ischemia in 12 patients (3.7%), acute renal failure in 7 patients (2.2%), and distal aortic rupture in 4 patients (1.2%). The pooled average of 1-year freedom from reintervention was 89.6%. Late reintervention was performed in 34 patients (10.4%), including thoracoabdominal aortic replacement for distal aortic dilation in 29 and thoracic endovascular aortic repair in 5. Proximal endoleak was managed with thoracic endovascular aortic repair in 3 patients.

### Negative Remodeling

After surgery at a mean of 13.4 months of follow-up negative remodeling occurred in 14.5% at TAAD patients (Table 2). And at a mean of 48.3 months of follow-up negative remodeling occurred in 32.1% (Table 3). At the latest follow-up CTA, complete aortic remodeling was observed at the distal surgical level in 78.7% (233 out of 296) and down to the mid-DA in 24.3% (72 out of 296) of patients.

**Table 2:** The first-time follow-up and morphologic characteristics data for patients with type A aortic dissection

Variable	Positive remodeling	Negative remodeling	*P* value
Cases	253	43	
Proportion (%)	85.5	14.5	
Follow-up time (mo)	13.11 ± 5.86	14.83 ± 6.13	.42
Age (y)	48.82 ± 15.31	50.8 ± 10.08	.06
Sex (male/female)	153/86	38/19	.26
Reoperation rate	1.2 (3)	7.1 (4)	<.01
Maximum diameter of residual dissection (mm)	33.6 ± 7.01	40.38 ± 10.18	<.01
No. of entry tears (n)	3.97 ± 1.27	3.81 ± 0.93	.08
Entry tears size (mm)	5.98 ± 2.01	5.54 ± 1.72	.02

Values are presented as mean ± standard deviation or % (n), unless otherwise noted.

**Table 3:** The second time follow-up and morphologic characteristics data for patients with type A aortic dissection

Variable	Positive remodeling	Negative remodeling	*P* value
Cases	201	95	
Proportion (%)	67.9	32.1	
Follow-up time (mo)	48.65 ± 15.56	46.12 ± 17.13	.42
Age (y)	52.04 ± 9.77	52.93 ± 10.13	.08
Sex (male/female)	127/74	68/27	.18
Death rate	7.0 (14)	13.7 (13)	<.01
Reoperation rate	4.5 (9)	24.2 (23)	<.01
Maximum diameter of residual dissection (mm)	36.84 ± 7.76	44.07 ± 9.32	<.01
No. of entry tears (n)	4.12 ± 1.58	3.76 ± 1.76	.03
Entry tears size (mm)	5.81 ± 1.48	5.17 ± 2.66	<.01
FET diameter (mm)	26.0 ± 1.5	26.5 ± 1.4	.025
FET length (mm)	120.4 ± 5.9	120.3 ± 5.1	.842

Values are presented as mean ± standard deviation or % (n), unless otherwise noted. *FET*, Frozen elephant trunk.

The positive remodeling patients share low rates of late death and distal reoperations of 7.0% (14 out of 201) and 4.5% (9 out of 201), compared with the negative remodeling patients (13.7% [13 out of 95] and 24.2% [23 out of 95]) (*P* < .01). Also, the maximum diameter of residual dissection of positive remodeling patients is smaller than in the negative remodeling patients (34.84 ± 7.76 vs 40.38 ± 10.18; *P* < .01). Maximal aortic sizes before discharge for negative remodeling patients were 43.2, 35.1, and 32.5 mm, and growth rates were 4.5 ± 1.52, 3.1 ± 1.14, and 3.5 ± 1.33 mm/year, at the level of diaphragm, celiac trunk, and renal artery, respectively, which is larger and expand more quickly than the patients with positive remodeling (*P* < .01) (Table 4). At the stent level, the TL diameter had significant increase (0.69 ± 0.23 cm; *P* < .01), the FL diameter had significant decrease (−0.86 ± 0.47 cm; *P* < .01), and the aorta diameter had significant decrease (−0.32 ± 0.43 cm; *P* < .05). At the distal end of the stent, the TL was still expanding, and the aortic expansion was quicker at the diaphragm level (4.5 ± 1.52 mm/year). For the aortic remodeling, at each level the negative remodeling was much more common below the renal artery level (23.5%).

**Table 4:** Maximal aortic sizes and growth rates at different evaluated levels

Variable	Positive remodeling	Negative remodeling	*P* value
Diaphragm level maximal aortic sizes	35.2 ± 4.17	43.5 ± 5.88	<.01
Celiac trunk level maximal aortic sizes	32.4 ± 5.13	34.5 ± 5.74	.11
Renal artery level maximal aortic sizes	29.8 ± 4.37	32.7 ± 6.23	<.01
Diaphragm level growth rate	2.3 ± 0.81	4.7 ± 1.36	<.01
Celiac trunk level growth rate	1.5 ± 0.44	2.9 ± 1.28	.06
Renal artery level growth rate	1.7 ± 0.79	3.3 ± 1.51	<.01

Values are presented as mean ± standard deviation.

### Entry Tears

Aortic remodeling rates based on the analysis of the dissected aortic segments were significantly influenced by the number of entry tears. Negative remodeling patients show fewer entry tears than the positive remodeling patients (3.46 ± 1.76 vs 4.02 ± 1.58; *P* = .05). Also, negative remodeling patients show smaller entry tear size (5.17 ± 2.66 vs 5.81 ± 2.48 mm; *P* < .01). The length of the interval between tears showed significant differences between the positive remodeling and the negative remodeling patients (*P* < .01), but this result was influenced by the number of tears.

Multivariate linear regression analysis showed that maximum diameter of residual dissection (odds ratio [OR], 1.87; 95% CI, 1.03-2.99; *P* = .054), maximum diameter of the aorta distal to the stent (OR, 2.36; 95% CI, 1.13-5.13; *P* = .025), and mean FL area distal to the stent before surgery (OR, 3.45; 95% CI, 1.08-6.89; *P* = .01) were associated with a significantly increased rate of negative remodeling. Increased mean TL area distal to the stent (OR, 0.44; 95% CI, 0.11-0.84; *P* = .042), increased number of intimal tears (OR, 0.37; 95% CI, 0.09-0.80; *P* = .026), and increased entry tears size (OR, 0.21; 95% CI, 0.07-0.74; *P* = .01) were associated with positive remodeling (Table 5). The distal FL area demonstrated the most pronounced effect on negative remodeling. However, within the scope of our case series, both the size and number of entry tears were identified as factors associated with negative remodeling, and these associations demonstrated statistical significance.

**Table 5:** Predictors of negative remodeling in multivariate linear regression analysis

Objective	Odds ratio	95% Cl	*P* value
Sex	1.31	0.31-3.07	.814
The maximum diameter of the aorta distal to the stent (mm)	2.36	1.13-5.13	.025
Mean false lumen area distal to the stent (cm^2^)	3.45	1.08-6.89	<.01
Mean true lumen area distal to the stent (cm^2^)	0.37	0.09-0.80	.026
No. of entry tears (n)	0.44	0.11-0.84	0042
Entry tears size (mm)	0.21	0.07-0.74	<.01

## Discussion

Our research demonstrates that apart from well-established risk factors such as MFS and maximum DA diameter, this study identifies the presence of a lesser and larger proximal entry tear as an additional predictor of mortality and the necessity for surgical intervention.^16^^,^^17^

The persistence of a patent FL in DA segments following surgical treatment for TAAD is common (64%-90%).^18^ Inadequate connection of the distal portion of the graft placed in the ascending aorta to the TL or the presence of secondary tears may explain the continued flow into the residual FL distally, despite complete surgical removal of the primary entry tear.^19^, ^20^, ^21^

In the present study, numbers, size, and proximal location of the entry tear were predictors of complications. Phantom studies have demonstrated that the larger the intimal tear in the proximal aorta, the greater the tendency to exacerbate TL collapse.^22^ Furthermore, Tsai and colleagues^13^ showed that systolic pressure in the FL, in comparison with the TL, falls with decreasing tear size. Therefore, increased pressure with smaller tear size will result in higher wall stress and risk of dilatation. They additionally suggested that proximal location of the entry tear was shown to cause a rise in FL diastolic pressure, thereby implying a tendency toward dilatation. Other studies suggested the possible predictive role of a proximal entry tear.^23^, ^24^, ^25^ Quint and colleagues^20^ assessed entry tear size and location by spiral CT in 52 patients with chronic aortic dissection, obtaining results similar to those of our series; the most common location was the proximal descending aorta.

Currently, the role of partial FL thrombosis in predicting aortic growth and its significance in aortic-related mortality remains incompletely understood. In our investigation, the number of patent entry tears may shed light on this issue because fewer entry tears between the TL and FL might promote FL thrombosis. As observed in our study, a reduced number of entry tears can impede blood outflow leading to sustained pressurization of the FL. This condition could induce thrombosis while altering normal laminar blood flow to a turbulent flow, thereby increasing wall stress.^26^ Combined with weakened vessel walls due to dissection, this process may facilitate aortic dilation.

We found that the number of entry tears observed on the initial CTA image may help identify patients at higher risk for aortic dilation who could benefit from early intervention. However, imaging of acute dissections can vary greatly depending on the phase of the cardiac cycle because the configuration of the aortic lumina and flap changes during systole and diastole. In this regard, dynamic imaging might provide the most accurate depiction of the condition. Nevertheless, static CT imaging remains the standard practice because dynamic imaging is not commonly used in clinical settings. Therefore, our study reflects the current standards of clinical practice. To address potential bias from reduced detection of entry tears, we assessed intraobserver and interobserver variability, which showed substantial agreement in identifying the number of entry tears.

Lastly, the average rate of aortic growth was lower in the infrarenal aorta compared with the thoracic aorta, but the negative remodeling was much more common below the renal artery level. This is due to the smaller diameter of the aorta below the renal artery. This difference may be due to lower hemodynamic forces in the abdominal aorta because previous studies have indicated that pulsatility is reduced in the abdominal aorta compared with the thoracic aorta.^27^ Additionally, FET can cover the proximal aortic tears, resulting in a lower rate of negative remodeling proximal to the stent.

The optimal management of patients discharged with persistent patent FL after treatment for acute aortic dissection remains controversial. Although short-term stability is common, the incidence of complications increases after 3 years, particularly in type B dissections.^28^, ^29^, ^30^ Closure of the entry tear, whether through surgical or endovascular means, promotes thrombosis of the FL and remodeling of the entire aorta.^31^ Treatment efficacy appears to be higher in the subacute phase compared with later stages when the aorta is more dilated and less elastic.^29^, ^30^, ^31^ Our findings suggest that imaging techniques during the subacute phase of aortic dissection may help identify patients at higher risk of complications. Besides a dilated aorta, poor clinical outcomes are more likely in those with proximal, fewer, and larger entry tears. Therefore, these patients may benefit from more intensive surveillance and treatment. These results underscore the importance of resecting or closing significant communications between the TL and FL in the distal ascending aorta or aortic arch in patients undergoing surgery for TAAD.

This study is limited by the lack of a control group of patients without MFS or a control group without FET, which means no definite conclusions can be drawn. Follow-up CTA was not available at all time points for all survivors, which may influence the power of our longitudinal analysis. And due to the resolution of the imaging data, some smaller entry tears could not be identified and counted, which influenced the conclusions. Our institution employs high-resolution CT with 0.625-mm slice thickness for vascular assessment, demonstrating sufficient resolution to detect most entry tears (all entry tears >1 mm). Although this imaging protocol inherently limits identification of subresolution microtears (a recognized technical constraint of the study design), systematic analysis of quantifiable parameters—including residual dissection size, number of identifiable entry points, and spatial distribution patterns—through standardized imaging protocols enables reliable postoperative risk stratification for negative remodeling, which maintains clinical significance. Furthermore, the observed influence of entry tear morphological characteristics on remodeling may be constrained within a specific range due to limitations inherent in our clinical case series. Extending these conclusions to broader populations will require additional investigation. We maintain that the apparent associations between entry tear size/number and negative remodeling should be interpreted cautiously, serving primarily as reference points that indicate an intriguing avenue for further research. We posit that the hemodynamic equilibrium resulting from these morphological variations constitutes the most critical determinant of postoperative remodeling. As our team's previous research has shown, morphological parameters affect the results of hemodynamics, thereby altering the prognosis of aortic disease.^32^ Consequently, we plan to validate this postulate more extensively in subsequent research through the development of hemodynamic models.

## Conclusions

Our results suggest that TAAD has a high rate of negative aortic remodeling in patients with MFS. Distal maximal aortic size, number of entry tears, and average entry tears size were all associated with death rate, reintervention rate, and the rate of negative aortic remodeling in TAAD patients with MFS.

## Conflict of Interest Statement

The authors reported no conflicts of interest.

The *Journal* policy requires editors and reviewers to disclose conflicts of interest and to decline handling or reviewing manuscripts for which they may have a conflict of interest. The editors and reviewers of this article have no conflicts of interest.
